# The effectiveness of self-management support interventions for men with long-term conditions: a systematic review and meta-analysis

**DOI:** 10.1136/bmjopen-2014-006620

**Published:** 2015-03-20

**Authors:** Paul Galdas, Jennifer Fell, Peter Bower, Lisa Kidd, Christian Blickem, Kerri McPherson, Kate Hunt, Simon Gilbody, Gerry Richardson

**Affiliations:** 1 Department of Health Sciences, University of York, York, UK; 2 NIHR School for Primary Care Research, Manchester Academic Health Science Centre, University of Manchester, Manchester, UK; 3 School of Health and Life Sciences/Institute for Applied Health Research, Glasgow Caledonian University, Glasgow, UK; 4 NIHR Collaboration for Leadership in Applied Health Research and Care (CLAHRC) Greater Manchester, Centre for Primary Care, Institute of Population Health, University of Manchester, Manchester, UK; 5 MRC/CSO Social and Public Health Sciences Unit, University of Glasgow, Glasgow, UK; 6 Centre for Health Economics, University of York, York, UK

**Keywords:** PRIMARY CARE, PUBLIC HEALTH

## Abstract

**Objectives:**

To assess the effectiveness of self-management support interventions in men with long-term conditions.

**Methods:**

A quantitative systematic review with meta-analysis.

**Data sources:**

The Cochrane Database of Systematic Reviews was searched to identify published reviews of self-management support interventions. Relevant reviews were screened to identify randomised controlled trials (RCTs) of self-management support interventions conducted in men alone, or which analysed the effects of interventions by sex.

**Review methods:**

Data on relevant outcomes, patient populations, intervention type and study quality were extracted. Quality appraisal was conducted using the Cochrane Risk of Bias Tool. Meta-analysis was conducted to compare the effects of interventions in men, women, and mixed-sex sub-groups.

**Results:**

40 RCTs of self-management support interventions in men, and 20 eligible RCTs where an analysis by sex was reported, were included in the review. Meta-analysis suggested that physical activity, education, and peer support-based interventions have a positive impact on quality of life in men. However, there is currently insufficient evidence to make strong statements about whether self-management support interventions show larger, similar or smaller effects in men compared with women and mixed-sex groups.

**Conclusions:**

Clinicians may wish to consider whether certain types of self-management support (eg, physical activity, education, peer support) are particularly effective in men, although more research is needed to fully determine and explore this.

**Strengths and limitations of this study**

- This is the first review to examine the moderating effect of sex in self-management support interventions.
- A substantial sample frame of 1887 potentially relevant studies (identified via 116 Cochrane reviews) were screened for eligibility against the inclusion criteria.
- The pragmatic nature of the search strategy, limiting to Cochrane reviews, means other relevant primary studies may have been missed, especially those published recently.

## Introduction

Around 15 million people in the UK suffer from long-term conditions (LTCs), defined as a health problem that cannot currently be cured but can be managed through medication, therapy and/or lifestyle modification, such as hypertension, asthma, diabetes, coronary heart disease and chronic kidney disease.^1^ ^2^ The figure is set to grow dramatically over the next 10 years, particularly those individuals living with three or more LTCs at once.^2^ The increasing burden of LTCs coupled with the financial pressures facing the NHS and other healthcare providers around the world is leading to a shift in healthcare delivery.^3^ Offering existing LTC care and services as currently configured will not be adequate if health and social care services are to be sustainable, person-centred, and appropriately target need while being resource efficient in the future.^2^ ^4^ Empowering and supporting the increasing number of people living with LTCs to develop their knowledge, skills and confidence to manage their own health has therefore become a key strategic objective of health providers.^5^ So called ‘supported self-management’ is a key mechanism for optimising quality, effectiveness and efficiency of LTC care because of the potential to improve health outcomes; help patients make better, more informed use of available healthcare support; and avoid interventions that are burdensome for patients, inappropriate to their needs, and inefficient for healthcare providers.^2^ ^6^ Delivered on a large scale, self-management support interventions have the potential to help reduce the overall costs of care without compromising patient outcomes.^7^

Despite growing evidence for their effectiveness, self-management support interventions are considerably limited in their ‘reach’; that is, the numbers of patients able or willing to access and engage with the intervention.^8–10^ The limited appeal and accessibility of existing self-management support services means only a minority of the eligible population currently benefit. Despite men being more likely than women to develop the most common and disabling LTCs such as chronic pulmonary disease, diabetes, and cardiovascular diseases,^11^ ^12^ less than one-third of participants engaging with some support services are men.^13–16^ This pattern of attendance is consistent with a growing body of research into men's identity and the management of illness which reveals that preventable risk factors, poor engagement in self-management, and reluctance to access existing health services account for a high proportion of mortality and morbidity in men.^12^ ^17–22^

Increasing recognition of men's gender-specific health needs have led to calls for tailored and targeted healthcare interventions,^11^ ^22^ including the recent European Commission report on the State of Men's Health in Europe.^12^ Self-management is one area where gender differences are likely to exist. If men show less benefit from current self-management interventions, there is a clear case for the development of tailored interventions that better meet their needs. However, if men do equally well or better in current interventions, the focus may move towards developing new methods to encourage men to attend such interventions. However, there is as yet no evidence either way, which means that there is no rigorous basis for evidence-based decisions to help clinicians and policymakers meet the specific needs of men with LTCs.

For these reasons, we conducted a systematic review to establish the relative effectiveness of self-management support interventions in men with LTCs. Our overarching question guiding the review was “How effective are self-management support interventions in men with LTCs?”. This was subdivided into two specific review questions:

1. How effective are self-management support interventions in men compared with women?
2. Are certain types of self-management support intervention more effective than others in men with LTCs?

Results from a parallel qualitative review of the acceptability and accessibility of self-management support interventions in men are reported elsewhere.^23^

## Methods

A systematic review and meta-analysis was conducted based on a protocol published on the PROSPERO database (Registration number: CRD42013005394).

### Electronic searches

We considered the incremental benefit from conducting a review of the primary literature to assess the comparative impact of interventions in men to be low, as the majority of relevant high-quality randomised trials of self-management support have already been assessed through existing Cochrane systematic reviews. We therefore took a pragmatic approach and limited our search to the Cochrane Database of Systematic Reviews (May Issue 5, 2013) using an electronic search strategy developed in conjunction with an information specialist (see table 1). We sought to identify relevant Cochrane systematic reviews of self-management support interventions (see online supplementary file 1), and identify individual trials of relevance that were included within these reviews that met the eligibility criteria described below.

**Table 1:** Search string for Cochrane Database of Systematic Reviews (CDSR)

Cochrane Library	
CDSR	records identified 3429
Search date:	18/07/2013
#1	MeSH descriptor: [Stroke] explode all trees
#2	MeSH descriptor: [Diabetes Mellitus] explode all trees
#3	MeSH descriptor: [Asthma] explode all trees
#4	MeSH descriptor: [Hypertension] explode all trees
#5	MeSH descriptor: [Depression] explode all trees
#6	MeSH descriptor: [Depressive Disorder] explode all trees
#7	MeSH descriptor: [Dementia] explode all trees
#8	MeSH descriptor: [Pulmonary Disease, Chronic Obstructive] explode all trees
#9	MeSH descriptor: [Renal Insufficiency, Chronic] explode all trees
#10	MeSH descriptor: [Irritable Bowel Syndrome] explode all trees
#11	MeSH descriptor: [Arthritis, Rheumatoid] explode all trees
#12	MeSH descriptor: [Arthritis, Psoriatic] explode all trees
#13	MeSH descriptor: [Spondylitis, Ankylosing] explode all trees
#14	MeSH descriptor: [Lupus Erythematosus, Systemic] explode all trees
#15	MeSH descriptor: [Low Back Pain] explode all trees
#16	MeSH descriptor: [Epilepsy] explode all trees
#17	MeSH descriptor: [Multiple Sclerosis] explode all trees
#18	MeSH descriptor: [Parkinson Disease] explode all trees
#19	MeSH descriptor: [Motor Neuron Disease] explode all trees
#20	MeSH descriptor: [Motor Neuron Disease] explode all trees
#21	MeSH descriptor: [Neoplasms] explode all trees
#22	MeSH descriptor: [Inflammatory Bowel Diseases] explode all trees
#23	MeSH descriptor: [Heart Diseases] explode all trees
#24	MeSH descriptor: [Skin Diseases] explode all trees
#25	MeSH descriptor: [Chronic Pain] explode all trees
#26	MeSH descriptor: [HIV] explode all trees
#27	MeSH descriptor: [Hepatitis] explode all trees
#28	MeSH descriptor: [Anxiety] explode all trees
#29	MeSH descriptor: [Psychotic Disorders] explode all trees
#30	(long* or chronic or long-term or long-standing or persistent or ongoing) near/2 (illness* or disease* or condition*)
#31	#1 or #2 or #3 or #4 or #5 or #6 or #7 or #8 or #9 or #10 or #11 or #12 or #13 or #14 or #15 or #16 or #17 or #18 or #19 or #20 or #21 or #22 or #23 or #24 or #25 or #26 or #27 or #28 or #29 or #30
#32	MeSH descriptor: [Self Administration] explode all trees
#33	MeSH descriptor: [Self Care] explode all trees
#34	"self care” or selfcare
#35	"self manag*” or selfmanag*
#36	"self monitor*” or selfmonitor*
#37	"self help” or selfhelp
#38	"self diagnos*” or selfdiagnos* or “self assess*” or selfassess*
#39	MeSH descriptor: [Self Medication] explode all trees
#40	"self medicat*” or selfmedicat* or “self remed*” or selfremed*
#41	"self treat*” or selftreat* or “self cure” or selfcure
#42	MeSH descriptor: [Self-Help Groups] explode all trees
#43	group near (support* or advice or advis* or monitor* or intervention* or train* or instruction or consult* or assist* or education or educate or information)
#44	peer near (support* or advice or advis* or monitor* or intervention* or train* or instruction or consult* or assist* or education or educate or information)
#45	"expert patient*”
#46	MeSH descriptor: [Telemedicine] explode all trees
#47	telemedicine or telecare or telenursing or telemonitor* or telehealth
#48	MeSH descriptor: [Remote Consultation] explode all trees
#49	(telephon* or remote or phone) near/2 (follow* or support or consult* or advice or advis* or intervention* or train* or instruction or assis* or educate or education or information or monitor*)
#50	"Action plan*”
#51	#32 or #33 or #34 or ‘35 or #36 or #37 or #38 or #39 or #40 or #41 or #42 or #43 or #44 or #45 or #46 or #47 or #48 or #49 or #50
#52	#31 and #51
#53	(man or man's or men or men's or male* or masculin* or gender* or sex difference* or sex factor*)
#54	#52 and #53

### Eligibility criteria

Our hypothesis was that sex would be a moderator of the effectiveness of self-management support interventions, and our specific review questions related to differential effects (whether self-management was more or less effective in men and women, and whether certain types of self-management were more effective in men). To answer these questions, we drew on the general literature on self-management support interventions, identifying within that literature the particular studies which answered our questions about differential effects.

To reflect this, we first present a general PICO (participants, interventions, comparisons and outcomes) formulation, identifying the scope of the literature on self-management interventions we used. We then present details of the different study types we identified within that literature to answer our specific questions.

#### Eligibility PICO

*Population:* Adults, 18 years or older, diagnosed with 1 or more of 14 ‘exemplar’ LTCs (asthma, diabetes, depression, hypertension, heart failure, chronic obstructive pulmonary disease (COPD), arthritis, chronic kidney disease, chronic pain, HIV, testicular cancer, prostate cancer, prostate hyperplasia and chronic skin conditions), in any setting.

*Intervention:* A self-management support intervention defined as an intervention primarily designed to develop the abilities of patients to undertake management of health conditions through education, training and support to develop patient knowledge, skills or psychological and social resources. There is no single agreed definition of what a self-management support intervention encompasses. We therefore developed a standardised criteria informed by the current literature on self-management support^7^ ^24^ which defined what we considered to be an intervention of relevance to the review (box 1).

*Comparison:* Usual care/non-self-management support intervention.

*Outcomes:* We extracted data on the effect of interventions on health status, clinical outcomes, health behaviour, healthcare use, self-efficacy, knowledge and understanding, communication with healthcare professionals (HCPs), and effects on family members/carers.

*Other criteria:* No date restrictions were imposed and only papers published in the English language were included. In instances where records were unobtainable, attempts were made to contact authors to request the information.

Within the studies meeting the PICO eligibility criteria, we restricted our review to randomised controlled trials (RCTs) identified via Cochrane systematic reviews of self-management support interventions. One researcher screened the titles and abstracts of retrieved records for Cochrane reviews that met the inclusion criteria. Following this, two researchers independently screened the full text of each potentially eligible Cochrane review article to identify reviews of relevance (see online supplementary file 1). Screening disagreements were resolved by a third researcher, as required.

Each relevant Cochrane review was then screened independently for eligible RCTs by two researchers. Eligibility of each RCT was checked using the study information presented within each Cochrane review prior to full papers being sourced. Full texts of potentially eligible RCTs were then screened independently by two researchers and disagreements were resolved by a third researcher, as required.

Box 1 **Criteria for defining a self-management support intervention**

The intervention should, through some means of education, training or support help people with long-term conditions by:

- Developing knowledge, skills, psychological or social resources relating to the management of their condition
- Adopting healthy life habits
- Helping individuals recognise the signs of deteriorating health status
- Planning actions to take at signs of relapse or exacerbation
- Knowing what resources are available and how to access them
- Developing skills for helping individuals adhere to a treatment plan
- Communicating effectively with health professionals and/or a support network
- Solving problems
- Identifying objectives, goals and developing action plans

We identified three groups of RCTs:

- RCTs investigating self-management support interventions with subgroup analyses analysing the effects of interventions in men and women
- RCTs investigating self-management support interventions in men with LTCs only
- RCTs investigating self-management support interventions in women with LTCs only, and in mixed-sex samples with LTCs (identified from the ‘parent’ Cochrane reviews that contained RCTs in men only) to derive comparison groups.

To answer the research question “How effective are self-management support interventions in men compared to women?”, we drew on RCTs investigating self-management support interventions with subgroup analyses, and comparisons of effects in RCTs investigating self-management support interventions in men, women and mixed-sex samples.

To answer the research question ‘Are certain types of self-management support intervention more effective in men with LTCs?’, we drew on effects in RCTs investigating self-management support interventions in men only.

### Data extraction

Two researchers piloted the data extraction sheet on a sample of papers prior to the main data extraction. Data were extracted by one researcher and independently checked for quality and accuracy by a second researcher. Data items, including study and population characteristics, intervention details and quality of life outcome measures, were extracted. Outcome data closest to 6 months follow-up was extracted for analysis, as measures around this time were the most frequently reported.

### Quality assessment

Study quality was assessed using the Cochrane risk of bias tool using the following domains: sequence generation, allocation concealment, blinding performance, incomplete outcome data, selective outcome reporting and other sources of bias. For pragmatic reasons, studies with mixed-sex and women-only samples that were used to derive comparison groups were assessed for quality based on the allocation concealment domain only. The purpose of the quality appraisal was to describe the quality of the evidence base, not as inclusion/exclusion criteria. RCTs containing gender subgroup analyses were assessed for quality using assessment criteria adapted from Pincus *et al*^25^ and Sun *et al*.^26^ ‘Yes’, ‘No’ or ‘Unclear’ were recorded as responses to the following quality appraisal questions:

1. Was the subgroup hypothesis considered a priori?
2. Was gender included as a stratification factor at randomisation?
3. Was gender one of a small number of planned subgroup hypotheses tested (≤5)?

### Coding intervention types

To facilitate the comparison of types of self-management support in the analyses, we generated a typology (see table 2) informed by the findings of two recent broader reviews of the effectiveness of self-management support^7^ ^24^ to categorise each intervention. Two members of the review team independently assigned categories to each self-management support intervention. Disagreements were resolved via discussion.

**Table 2:** Typology of self-management support interventions

Self-management support intervention category	Description
Physical activity	Includes any study where physical activity occurs that is, a class or self-directed home-based work. Those containing purely advice or promotion should be captured under education
Education	Includes any study where education is taught or educational materials are provided to patients. This may include skills training and dietary or physical activity guidance
Peer support	Peer support provided by ‘peers’ that is, other patients. This may be in the form of a ‘buddy’ system or through interaction at support groups. HCP support may be captured under HCP monitoring and feedback
Psychological interventions	Includes professional counselling or therapy
HCP monitoring and feedback	Support in the form of health monitoring and/or feedback on a regimen/promoted lifestyle change. Excludes support provided by peers which should be captured under peer support
Action plans	A plan of actions or responses agreed with and used by the patient in response to particular situations for example, symptom exacerbation, dose adjustment according to symptoms
Financial incentives	Includes any intervention where financial barriers are removed or incentives are used to motivate patients to follow a particular intervention or lifestyle change

HCP, healthcare professional.

### Data analysis

Meta-analysis, where feasible (reported in the following sections) was conducted using Review Manager V.5.2. Data were extracted, analysed, and presented as standardised mean difference to account for the different instruments used. As a guide to the magnitude of effect, we categorised an effect size of 0.2 representing a ‘small’ effect, 0.5 a ‘moderate’ effect, and 0.8 a ‘large’ effect.^27^ A random effects model was used to combine study data and statistical heterogeneity was assessed with the I^2^ test, with ‘low’ heterogeneity set at ≤25%, ‘moderate’ 50% and ‘high’ 75%.^28^ In instances where studies contained multiple intervention groups, each group was extracted and analysed independently, dividing the control group sample size to avoid double counting in the analysis.

The results of three analytical approaches are reported in this paper:

Analysis #1: ‘Moderating effect of sex in individual trials of self-management support’

We identified individual RCTs of self-management support interventions where an effect of sex had been reported. We sought to extract relevant data on the direction and size of moderating effects in secondary analysis (ie, whether men show larger, similar or smaller effects than women), and assess these effects in the context of relevant design data, such as sample size, and the quality of the secondary analysis.

Analysis #2: ‘Effectiveness of self-management support interventions in men compared with women and mixed-sex sub-groups’

**Figure 1:**
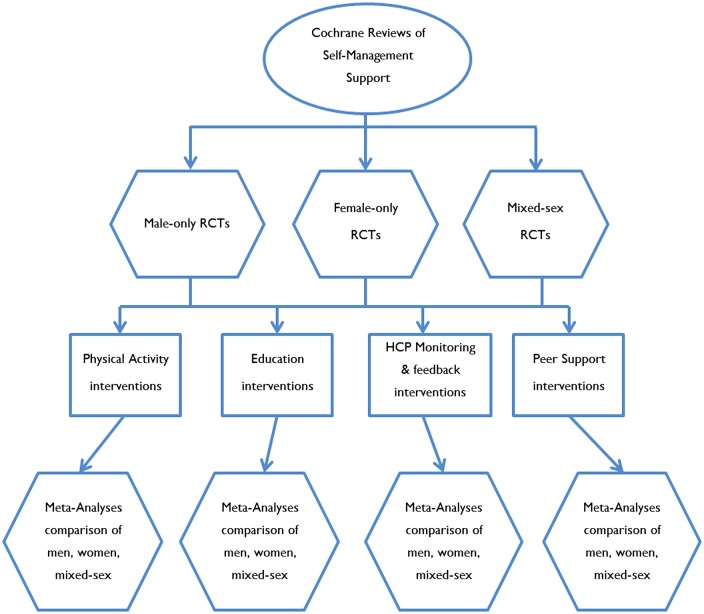
Analysis #2 (HCP, healthcare professionals; RCT, randomised controlled trial).

Studies with mixed-sex or women-only samples were identified from the ‘parent’ Cochrane reviews that contained trials of self-management support in men to derive comparison groups. Data were pooled according to broad intervention type (see figure 1) to allow us to determine whether broad types of self-management support interventions show larger, similar or smaller effects in men compared with women and mixed-sex populations. Limitations in the data meant we were only able to conduct analyses on physical activity, education, peer support, and HCP monitoring and feedback interventions.

We report the effect size (together with significance and 95% CI) of self-management support in each sex ‘sub-group’ (men-only, mixed-sex, women-only). We conducted analyses to test whether interventions showed significantly different effects in sex subgroups. In many cases the number of studies and/or sample size was also small which may have limited the power to detect important differences. Drawing comparisons across trials also has some limitations, in that such comparisons do not have the protection of randomisation and as such findings may be confounded by other differences between studies other than sex mix, such as trial quality. For each analysis we presented data on the comparability of the included studies, including the mean age of participants in the intervention group and the quality of the study (using allocation concealment as an indicator of quality).

Analysis #3: ‘Effectiveness of self-management support interventions in men’

**Figure 2:**
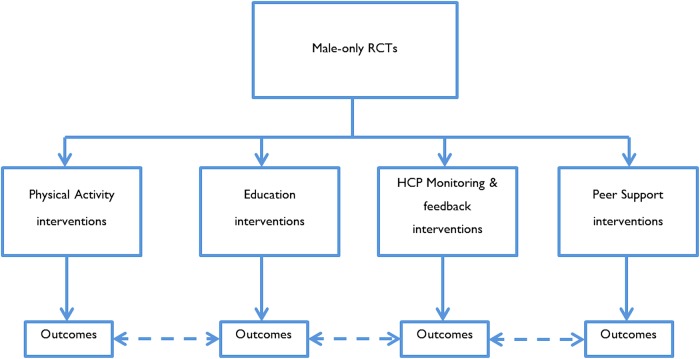
Analysis #3 (RCT, randomised controlled trial).

We carried out a meta-analysis on trials of self-management support conducted on samples of men alone. We did this by broad intervention type—physical activity, education, peer support, and HCP monitoring and feedback—and compared effects between intervention types (see figure 2). This allowed us to determine whether the presence of certain components of self-management support were associated with larger effects.

## Results

**Figure 3:**
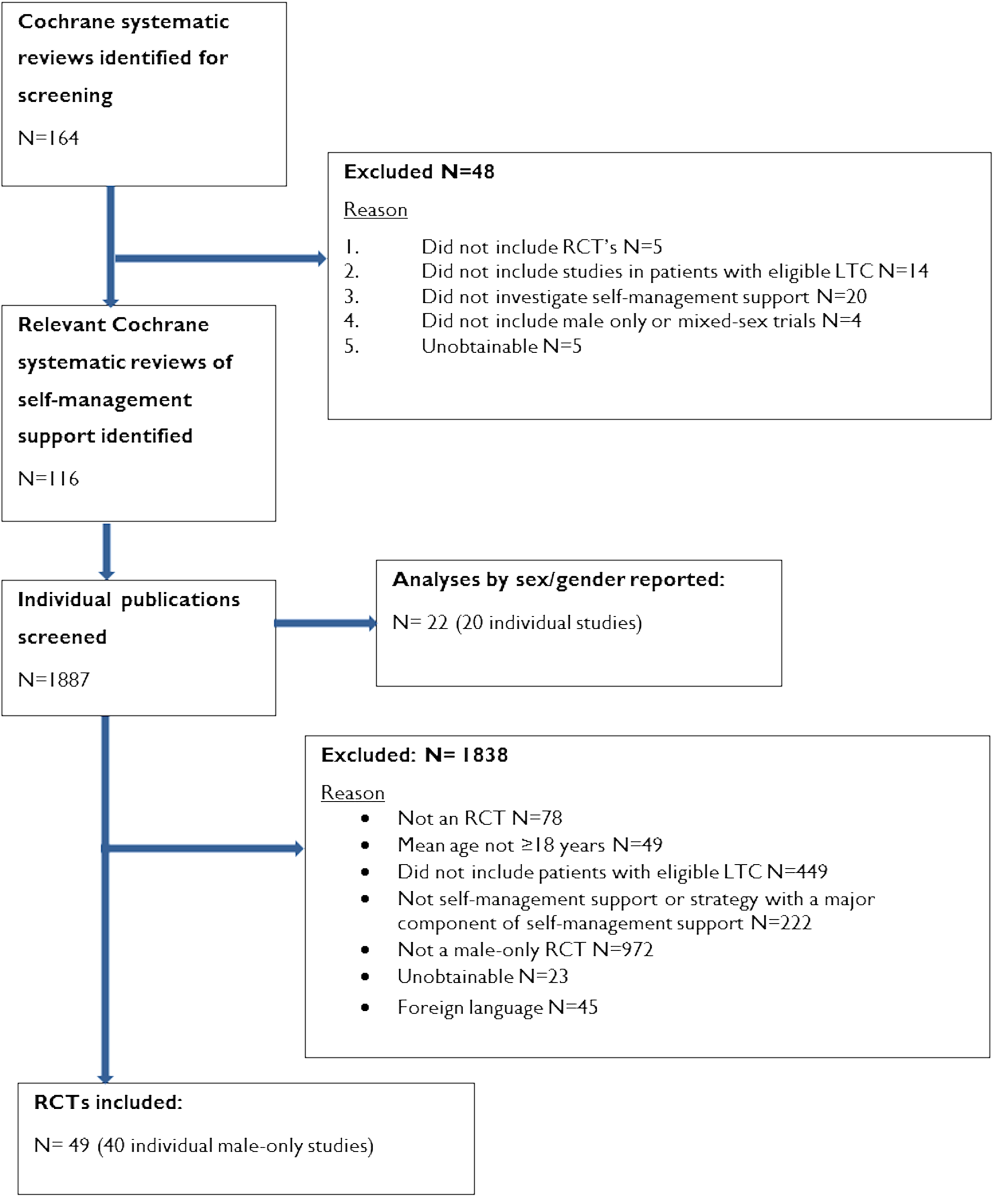
Exclusion of criteria and paper selection flow chart (LCT, long-term condition; RCT, randomised controlled trial).

The PRISMA flow diagram detailing publication inclusion and exclusion numbers is presented in figure 3. Study characteristics of all included studies are provided in online supplementary file 2.

### Study characteristics: RCTs of self-management support in men

The search identified 164 Cochrane systematic reviews, of which 116 were eligible for inclusion. A total of 1887 individual publications within the included Cochrane reviews were screened, resulting in the inclusion of 40 RCTs of self-management support in samples of men alone (see figure 3). The exact number of RCTs included in each meta-analysis is reported under each analysis subheading.

The majority of men-only studies were conducted in the USA (n=23), other locations were Europe (n=11), Canada (n=5) and Poland (n=1). Disease types in the recruited populations included prostate cancer (n=15), hypertension (n=6), COPD (n=6), heart failure (n=4), diabetes type II (n=3), diabetes unspecified type (n=1), arthritis (n=1) and testicular cancer (n=1). One multimorbidity study recruited obese men with type II diabetes and chronic kidney disease. The age of participants ranged from 25 to 89 years and, where reported, ethnicity was predominantly Caucasian.

A total of 51 distinct self-management support interventions were reported across the 40 included men-only studies. Physical activity (n=16), education (n=36), peer support (n=17) and HCP monitoring and feedback (n=25) were the most frequently reported major components of these interventions.

Group interventions (n=20), interventions at an individual level (n=23) or a mixture of both approaches (n=6) were employed. It was unclear what approach was used in two studies. Duration of interventions was variable across studies ranging from hours to over 12 months.

### Study characteristics: RCTs of self-management support in women or mixed-sex samples

A total of 32 mixed-sex or women-only trials were identified from Cochrane reviews with included men-only studies with data suitable for meta-analysis.^27^ ^29–59^ The majority of studies recruited patients with cancer (n=25 various diagnosis) with the remaining recruiting patients with chronic kidney disease (n=3), COPD (n=2) and HIV (n=2). Studies were largely conducted in the USA (n=16), other countries included Canada (n=4), UK (n=2), India (n=2), Denmark (n=2), Netherlands (n=1), Sweden (n=1), Norway (n=1), Greece (n=1), Taiwan (n=1) and unclear (n=1).

### Study characteristics: RCTs of self-management support with subgroup analysis of the effects of interventions in men and women

A total of 20 mixed-sex RCTs included a subgroup analysis of the effects of interventions in men and women. The majority of studies recruited patients with diabetes (n=7) with the remaining recruiting patients with chronic pain (n=6), heart failure (n=3), hypertension (n=1), dysthymia (n=1), osteoarthritis (n=1) and HIV (n=1). Nine studies were conducted in the USA, other countries included Finland (n=6), Sweden (n=1, Norway (n=1), Germany (n=1), Argentina (n=1) and Africa (n=1).

### Risk of bias

**Figure 4:**
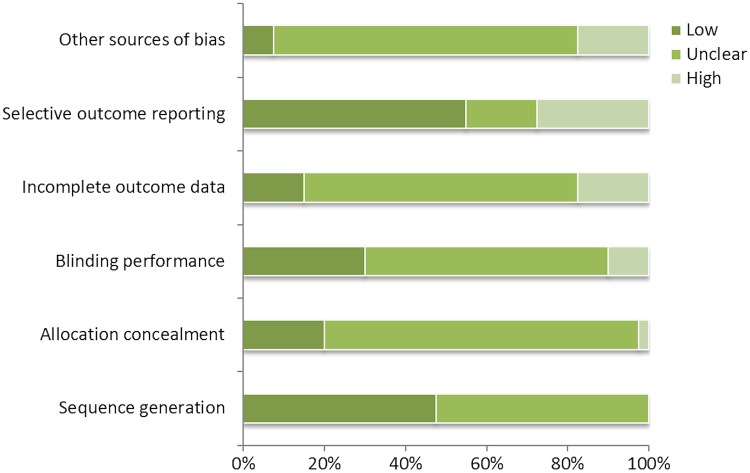
Summary of Cochrane risk of bias for male-only trials.

Trials involving samples of men alone were often poorly reported, making judgments of quality difficult. With the exception of selective outcome reporting, the most frequent rating for all domains was an unclear risk of bias. For the selective outcome reporting domain, a low risk of bias was most frequently assigned (see online supplementary file 2 and figure 4). In analysis #2, quality assessment of studies involving mixed-sex and women-only samples was based on allocation concealment. Of the 32 mixed-sex or women-only RCTs, 12 were found to have a low risk of bias and 20 were unclear in relation to allocation concealment bias (see online supplementary file 2). Quality assessment findings of trials conducting a subgroup analysis of the effects of interventions on men and women are reported in the table of study characteristics (see online supplementary file 2).

### Results of meta-analyses

Analysis #1: Moderating effect of sex in individual trials of self-management support

A total of 20 trials were identified where an effect of sex had been reported for intervention and control groups in secondary analyses. However, we found that data within these were inconsistently and often inadequately reported for the purpose of this review, and therefore did not allow for comparison between effect sizes in men and women.

Analysis #2: Effectiveness of self-management support interventions in men compared with women and mixed-sex subgroups

A total of 17 trials of self-management support in men, 15 trials of self-management support in women, and 17 trials with mixed-sex samples were used in the meta-analysis of the comparative effects of different types of self-management support intervention in men, women and mixed-sex groups with a range of LTCs (table 3). For physical activity, education or peer support-based interventions, small-to-moderate effects which reached statistical significance were consistently observed for men in health-related quality of life (HRQOL) outcomes. A similar pattern was evident for fatigue outcomes (with the exception of peer support which was non-significant), in that men consistently demonstrated significant benefits, although the largest effect size was not always observed in the men-only group.

**Table 3:** Results of meta-analysis (analysis #2)

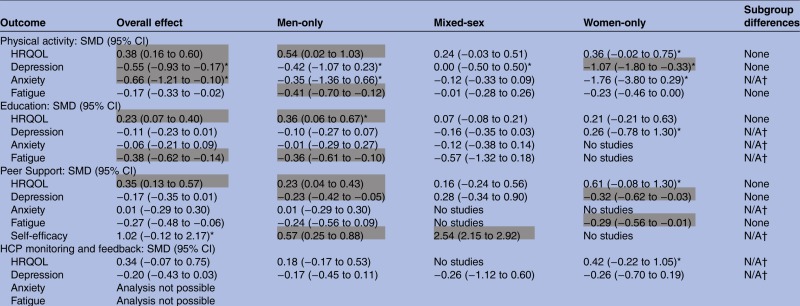

Cells highlighted in grey indicate a significant effect size was determined that is, the 95% CIs do not contain zero and the effect size is greater or equal to 0.2 (ie, at least a small effect).

*Indicates where heterogeneity is high.

†In cases were individual subgroups were non-significant or had insufficient trials numbers a test for subgroup differences was not performed.

HCP, healthcare professional; HRQOL, health-related quality of life; SMD, standardised mean difference.

For depression outcomes, only men-only groups receiving peer support-based interventions achieved a small but statistically significant effect. Larger significant effects were evident for women-only groups for physical activity and peer support interventions.

No significant effects on anxiety outcomes were observed in any analyses; however, data was limited for this outcome. No significant effects were observed on any outcomes for HCP monitoring and feedback interventions but, again, data was limited for this intervention type.

Analysis #3: Effectiveness of self-management support interventions in men

The analysis explored whether self-management support interventions with particular components were consistently more or less effective than those without those components in trials involving men with a range of LTCs (table 4). A total of 14 trials of self-management support in men reported data amenable to meta-analysis. Limitations in the data meant we were only able to conduct analyses on interventions with a physical activity; education; peer support; and/or a HCP monitoring and feedback component.

**Table 4:** Results of meta-analysis (analysis #3)

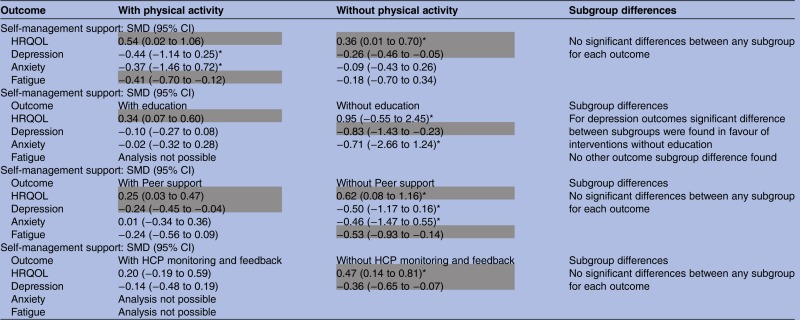

Cells highlighted in grey indicate a significant effect size was determined that is, the 95% CIs do not contain zero and the effect size is greater or equal to 0.2 (ie, at least a small effect).

*Indicates where heterogeneity is high.

HCP, healthcare professional; HRQOL, health-related quality of life; SMD, standardised mean difference.

Physical activity interventions had greater effects on HRQOL, depression, anxiety and fatigue outcomes than those without. Conversely, education, peer support, or HCP monitoring and feedback intervention effects for HRQOL, depression and anxiety were greater in the absence of these respective components, although the effect was not always significant. In relation to education interventions, effects were only statistically significant for interventions *with* education for HRQOL outcomes and *without* education for depression outcomes. The effect size was large for depression outcomes and a subgroup test for differences was significant, indicating the positive effect on depression outcomes was associated with the characteristics of those interventions *without* an education component.

In relation to peer support interventions, effects were statistically significant for interventions *with* or *without* a peer support component for HRQOL outcomes, with a greater effect size found for interventions without peer support. Significant effects were also observed for interventions *with* peer support for depression outcomes. Although the effect size was small, peer support was the only intervention type to exhibit a statistically significant effect in depression outcomes. A moderate and significant effect was also observed for interventions *without* peer support for fatigue outcomes. For HCP monitoring and feedback outcomes, only those interventions *without* this component statistically benefited HRQOL and depression outcomes.

## Discussion

### Principal findings

The question of whether the benefits of self-management support are moderated by sex is clearly of relevance to clinicians and policymakers. We identified 116 eligible reviews and 1887 individual publications. However, there were only 40 RCTs of self-management support interventions involving men alone, and a consistent failure to report appropriate subgroup analyses that might have enabled a rigorous assessment of differential effects.

Overall, our analyses suggest that physical activity, education, and peer support-based interventions may be particularly beneficial for improving HRQOL in men. However, there is currently insufficient evidence to make strong statements about whether men show larger, similar or smaller effects in self-management support interventions compared with women and mixed-sex groups.

### Strengths and limitations

The innovative approach to analysis used in this review is an obvious strength. The review questions were examined using multiple methods (subgroup analyses within trials, comparisons across trials using different sex mix, and analysis within trials of men alone) and levels of abstraction to see if there were any key trends across the analyses. To the best of our knowledge, this is the first time this approach has been used to examine the moderating effect of sex in self-management support interventions. An additional strength of the review lay in the breadth of our search, generating a substantial sample frame of 1887 potentially relevant studies (identified via 116 Cochrane reviews) that were screened for eligibility against our inclusion criteria.

However, the review has some inherent limitations. The pragmatic nature of the search strategy, limiting to Cochrane reviews, means other relevant primary studies may have been missed, especially those published recently. Foreign language papers were also not translated. However, it seems unlikely that these additional sources would have provided significant numbers of new studies that would have had a profound impact on the results of the syntheses.

In some analyses, the number of studies and/or sample size was small which may have limited power to detect important differences. Furthermore, as noted earlier, comparisons *across* trials such as those made in analysis #2 do not have the protection of randomisation, and there may be differences between the studies included in each sex subgroup which account for differences in effects between subgroups.

Clinical and statistical heterogeneity were also evident in some cases, and caution must be taken in interpreting results in these instances. Reasons for heterogeneity were explored where possible, although limitations in reporting and small numbers of included studies made detailed exploration difficult. We did intend to distinguish between men-only studies with standard care/waiting list controls comparators and those with active comparators. However, only one study in the men-only meta-analysis had an ‘active’ comparator, and this compared supervised physical activity with unsupervised (and thus included an ‘active’ comparator of lower intensity that was not self-management support), and it seems unlikely this would have had a substantive impact on the findings.

### Implications for clinicians and policymakers

Clinicians and those involved in designing interventions may wish to consider whether certain types of self-management support are particularly effective in men. In the existing data that is amenable to analysis, evidence of effects on quality of life point towards men benefitting the most from physical activity, education, and peer support-based interventions; however, more research is needed to fully determine and explore this.

### Unanswered questions and future research

Clearly, further primary research is needed to examine which models of service delivery are most effective in providing self-management support to men (and women). Any intervention developed should be theory-led, and our review findings point towards some broad components of interventions which could act as a starting point for testing the ‘active ingredients’ successful at promoting self-management in men. Parallel qualitative research is also indicated to test out theory and develop our understanding of what makes interventions, and their ‘active ingredients’, accessible and acceptable for men with LTCs.

Men's engagement with self-management support interventions is complex and contextually dependent,^23^ and traditional approaches to measuring effectiveness such as those reported here offer a limited insight into why an intervention works or does not work when applied in different contexts.^60^ A study drawing on realist principles^60^ might therefore be one method of analysis which has utility in the future.

Our ability to answer our review questions were hampered by a lack of consideration and/or inadequate reporting of sex as a moderator of outcome data in primary studies. Few studies provided outcome data separately for men and women. There is a need for researchers to consistently consider sex in their analyses and provide consistent and comprehensive reporting of outcomes by sex. Access to primary databases through archives or the online supplementary material functions of online publications may be one way of facilitating such analyses, and concerns about power and precision may be managed through adoption of appropriate statistical techniques.^61^ Support interventions also need to be clearly and consistently described by researchers using a shared language. We recommend that researchers clearly report on whether an intervention was intended to target a specific behaviour change and report adequate detail to allow for coding with the behaviour change techniques taxonomy,^62^ where applicable.
